# MicroRNA-31 suppresses medulloblastoma cell growth by inhibiting DNA replication through minichromosome maintenance 2

**DOI:** 10.18632/oncotarget.2043

**Published:** 2014-05-30

**Authors:** Yucui Jin, Anwen Xiong, Ziyu Zhang, Sanen Li, Huijie Huang, Ting-ting Yu, Xiumei Cao, Steven Y. Cheng

**Affiliations:** ^1^ Department of Developmental Genetics, School of Basic Medical Sciences, Nanjing Medical University, Nanjing, Jiangsu, China; ^2^ Department of Oncology, Changhai Hospital, Second Military Medical University, Shanghai, China; ^3^ Shanghai Institute of Immunology, Shanghai Jiao Tong University School of Medicine, Shanghai, China

**Keywords:** medulloblastoma, miR-31, tumorigenesis, MCM2, tumor cell growth

## Abstract

Medulloblastoma is an aggressive childhood brain tumor with poor prognosis. Recent studies indicate that dys-regulation of microRNA expression plays important roles in tumorigenesis. By comparing microRNA levels between mouse medulloblastoma and normal cerebellar tissues, we identified a set of down-regulated microRNAs including miR-31. Here, we show that the genomic region surrounding human miR-31 at 9p21.3 is frequently deleted in many solid tumor cell lines, and reintroducing miR-31 into DAOY cells, a line of human medulloblastoma cells devoid of miR-31, strongly suppresses cell growth, causes cell cycle arrest at the G1/S boundary, and inhibits colony formation in vitro and xenograft tumorigenesis in nude mice. Global gene expression profiling of mouse medulloblastomas and bioinformatics analyses of microRNA targets suggest that minichromosome maintenance complex component 2 (MCM2) is a likely target gene of miR-31 in suppressing cell growth. We demonstrate that miR-31 inhibits MCM2 expression via its 3'-untranslated region, that knockdown of MCM2 in DAOY cells leads to a degree of growth inhibition comparable to that by miR-31 restoration, and that overexpression of miR-31 reduces the chromatin loading of MCM2 at the point of G1/S transition. Taken together, these data indicate that miR-31 suppresses medulloblastoma tumorigenesis by negatively regulating DNA replication via MCM2.

## INTRODUCTION

Most cancers arise in old ages as the consequence of accumulative genetic lesions in the genome [1]. Recent cancer whole genome sequencing studies indicate that, on average, human solid adult tumors can have mutations affecting over 100 genes [2]; however, most of these cancer associated genes are altered by noncancer-causing passenger mutations. In contrast, pediatric cancers, which occur early in life and are rare, have much fewer genetically altered genes, making them ideal places for finding cancer-causing driver mutations.

Medulloblastoma is a highly malignant tumor of the cerebellum and the most common brain tumor in children [3]. Recent mouse model studies have identified two populations of cells, namely the granule neuron progenitors in the external germinal layer and the multipluripotent neural stem cells likely from the dorsal brain stem [4], as the cellular origin of medulloblastoma. Molecularly, medulloblastoma has been classified into four subtypes based on global gene expression profiling and DNA copy number analyses; these include tumors with aberrant regulation in the WNT and Sonic Hedgehog (SHH) pathways as well as two other less defined Group 3 and Group 4 tumors. These four subgroups share partial characteristics with the WHO stratification, but each has distinct clinical, demographic, and biological features [5-8]. The WNT subgroup has a relatively benign clinical presentation and a favorable prognosis under current treatment regimens [9], whereas Group 3 and 4 can be invasive and metastatic [10]. Currently, mutations in tumor suppressors PATCHED and SUFU of the SHH pathway as well as aberrant activation of CTNNB1, MYC, and the 17-92 cluster of microRNAs have been causatively linked to the etiology of medulloblastoma [6]. Despite these impressive advances, the confirmed genes only account for a small percentage of targets for all genetic lesions that lead to medulloblastomas [2].

MicroRNAs are a class of genomically encoded small RNAs of about 22 nucleotides in length that negatively regulate gene expression through imperfect base pairing with recognition sequences in the 3'-untranslated regions (3'UTR) of target mRNAs, resulting in either mRNA degradation or interference with translation [11]. MicroRNAs are important regulators of essentially all normal cellular processes [12-16] as well as pathogenesis of many diseases including cancer. Depending on the function of target genes, microRNAs can be oncogenes or tumor suppressor genes [17, 18]. Recent studies have implicated several microRNAs in the tumorigenesis of medulloblastoma, including miR-124 acting as a tumor suppressor and the miR-17/92 cluster as oncogenes in the SHH group of medulloblastomas [19-21].

Here, we report that miR-31, selected from our deep sequencing analysis of the spontaneous *Ptch*^+/-^ mouse models, is a potent inhibitor of medulloblastoma cell growth. MiR-31 has emerged as an important player in a number of cancers acting as a potent suppressor of proliferation in ovarian, prostate and breast cancer cells [22-24]. Our results indicate that miR-31 also suppresses tumor initiation by targeting minichromosome maintenance complex component 2 (MCM2), thus providing a check point control over initiation of DNA replication.

## RESULTS

### Down-regulation of miR-31 in mouse and human medulloblastoma cells

**Figure 1:**
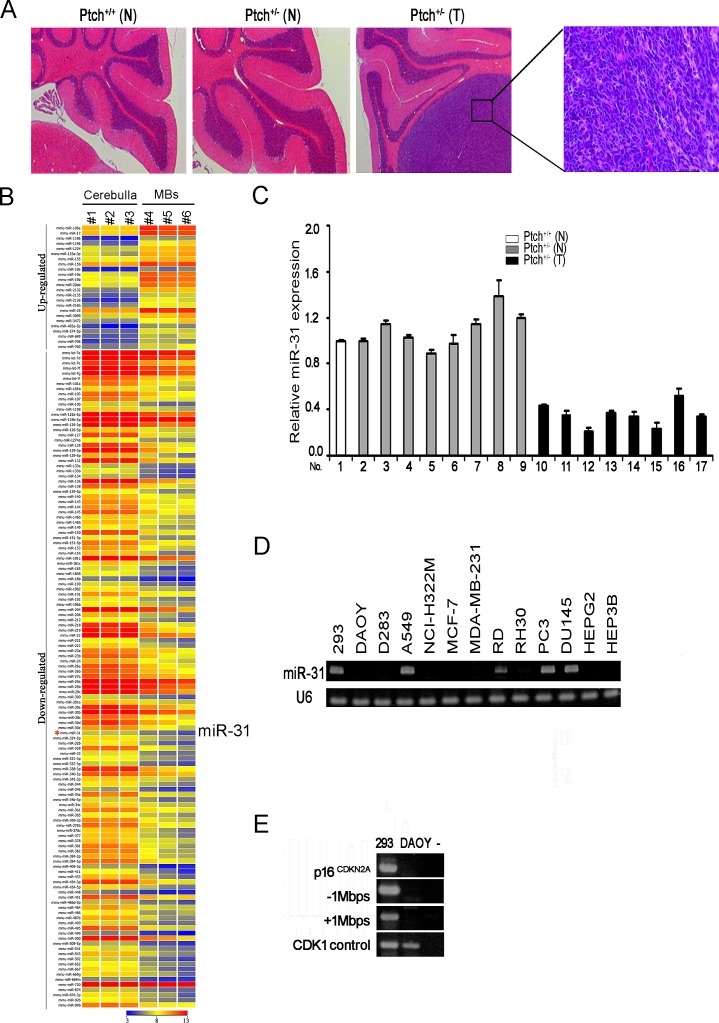
MiR-31 is down-regulated in mouse and human medulloblastoma cells (A) Hematoxylin and Eosin (H&E) staining of sagittal sections of normal cerebellar and medulloblastoma tissues from Ptch^+/-^ mice. N, normal cerebella. T, medulloblastoma. (B) Heat map of differential miRNA expression by NanoString nCounter analysis of Ptch^+/-^ mouse medulloblastoma and age-matched normal cerebellar tissues. (C) Quantitative RT-PCR analysis of miR-31 using RNAs extracted from normal cerebellar and medulloblastoma tissues. Results were normalized to *U6*. Error bars indicate standard deviation. (D) Stem-loop RT-PCR analysis of miR-31 using RNAs extracted from HEK293 cells, human medulloblastoma cell lines DAOY and D283, and lung (A549, NCI-H322M), breast (MCF-7, MDA-MB-231), liver (HEPG2, HEP3B), prostate (PC-3, DU-145), and soft tissue rabdomyosarcoma (RD, RH30) cancer lines. U6 was used as an internal control. (E) Genomic PCR analysis of the p16^CDKN2A^ locus and its surround areas in DAOY cells. Genomic DNA of 293 cells served as a positive control. “-” denotes water control.

Ptch^+/-^mice housed in our animal facility developed medulloblastomas spontaneously with a frequency of approximately 17% within 7 months of age. The cells in these tumors appeared small with dark carrot-shaped nuclei and sparse cytoplasm (Figure 1A). To identify microRNAs contributing to medulloblastoma tumorigenesis, we performed NanoString nCounter analysis of three spontaneous medulloblastomas from Ptch^+/-^ mice and compared them to three age-matched normal cerebella. Total RNAs from medulloblastoma and normal cerebellar tissue of Ptch^+/-^ mice were analyzed using NanoString nCounter miRNA expression assay containing probes for 578 mouse miRNAs ^[27]^. Based on a 3-fold or greater difference in mean values in medulloblastoma samples relative to normal cerebellar tissue (p<0.01), we found that 152 miRNAs were significantly differentially expressed (Figure 1B and Supplementary Table 1). Twenty-four miRNAs were up-regulated and 128 miRNAs showed down-regulation in medulloblastoma. One of these microRNAs is miR-31 that has been shown previously to play an important role in metastasis [22, 24], and also reported as being down-regulated in human medulloblastomas compared to normal cerebella [28]. To validate the NanoString data, we examined miR-31 expression in an enlarged series of normal cerebellar (n=9) and medulloblastoma tissues (n=8), and found that miR-31 was indeed down-regulated in all medulloblastoma samples compared to the normal controls (Figure 1C).

To determine if down-regulation of miR-31 is a general phenomenon associated with tumorigenesis, we examined miR-31 expression in two established lines of human medulloblastoma cells DAOY and D283, and a number of lung, breast, liver, prostate, and soft tissue cancer lines including A549, NCI-H322M, MCF-7, MDA-MB-231, HEPG2, HEP3B, PC-3, DU-145, RD, and RH30. The results indicated that except for A549, RD, PC-3 and DU-145, expression of miR-31 was completely abolished in the rest group (Figure 1D), suggesting that miR-31 might impose a hindrance to cell growth that must be overridden during tumorigenesis. In the human genome, miR-31 is located at 9p21.3, in the vicinity of tumor suppressor, p16*^CDKN2A^,* which is frequently deleted in solid tumor cells [29, 30]. Genomic PCR of DNA isolated from DAOY cells indicated a large region at the p16*^CDKN2A^* locus extending at approximately 1 Mbps both up and down stream, including the miR-31 gene (Figure 1E). It is possible that miR-31 was deleted fortuitously along with the p16*^CDKN2A^* locus, or alternatively, miR-31 may have a tumor suppressor function of its own.

### Restoring miR-31 expression inhibits DAOY cell growth, colony formation and xenograft tumorigenesis

**Figure 2:**
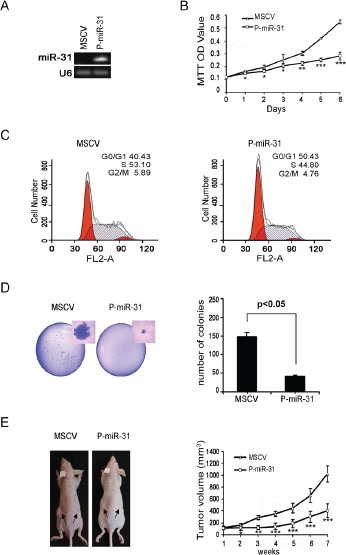
Restoring miR-31 expression inhibits DAOY cell growth, colony formation and xenograft tumorigenesis (A) DAOY cells were transfected with constitutive miR-31 expression vector, and subsequently selected in puromycin. Monoclonal cell clones were collected and subjected to stem-loop RT-PCR analysis. DAOY cells carrying the empty MSCV vector were used as the control. (B) MiR-31 expressing DAOY cells and vector control cells were seeded onto a 96-well plate. Cell viability was assessed in six consecutive days by MTT assay. (C) Histograms of distribution of DNA content in vector control and miR-31 expressing DAOY cells by flow cytometry. The percentages of cells in G1, S and G2 phase of the cell cycle were determined by analysis with the Multicycle computer software. (D) Results of colony formation assay are presented. 500 cells seeded onto a p60 plate and allowed to grow until visible colonies appeared. Colonies were stained with Giemsa. The representative figure from each group photographed (on left panel). Statistical analysis of the colony number from each group was shown on the right panel. (E) Nude mice were injected subcutaneously with vector control and miR-31 expressing DAOY cells as described in the Material and Methods section. The representative figure of mice from each group photographed at time of sacrifice (on left panel). Statistical analysis for tumor volumes from control (MSCV) and miR-31 expressing group (P-miR-31) at different time point was shown on right panel.

To determine if miR-31 indeed possesses a tumor suppressor function, we generated a pool of stable DAOY cells, P-miR31 cells, carrying genomically integrated copies of MSCV transcription units that constitutively express miR-31 (Figure 2A). Relative to those carrying the empty MSCV viral vector, the MSCV DAOY cells, P-miR-31 DAOY cells grew much slower by daily cell counts (data not shown) and had reduced rate of metabolism as evident by quantification using tetrazolium dye, 3-(4,5-dimethylthiazol -2-yl)-2,5-diphenyl tetrazolium bromide (MTT) (Figure 2B). FACs analysis indicated that 50.43% of P-miR-31 DAOY cells accumulated in the G1 phase, comparing to 40.43% of MSCV DAOY cells, and the percentage of P-miR-31 DAOY cells in the S phase was reduced correspondingly to 44.80% from 53.10% of the control cells (Figure 2C). These data suggest that reintroducing miR-31 likely restored a G1 to S phase check point control. P-miR-31 DAOY cells also exhibited reduced capacity to support colony formation when cultured at low density, as both the number and the size of foci were markedly lower compared to those exhibited by the MSCV DAOY cells (Figure 2D). When injected subcutaneously into the flanks of nude mice, P-miR-31 DAOY cells sustained a much lower xenograft tumor growth compared to the MSCV DAOY cells, as evident by weekly measurements of the tumor volumes (Figure 2E). Thus, our experiments demonstrated that miR-31 indeed possesses the ability to suppress medulloblastoma tumor cell growth both *in vitro* and *in vivo*.

### MCM2 is a target of miR-31

**Figure 3:**
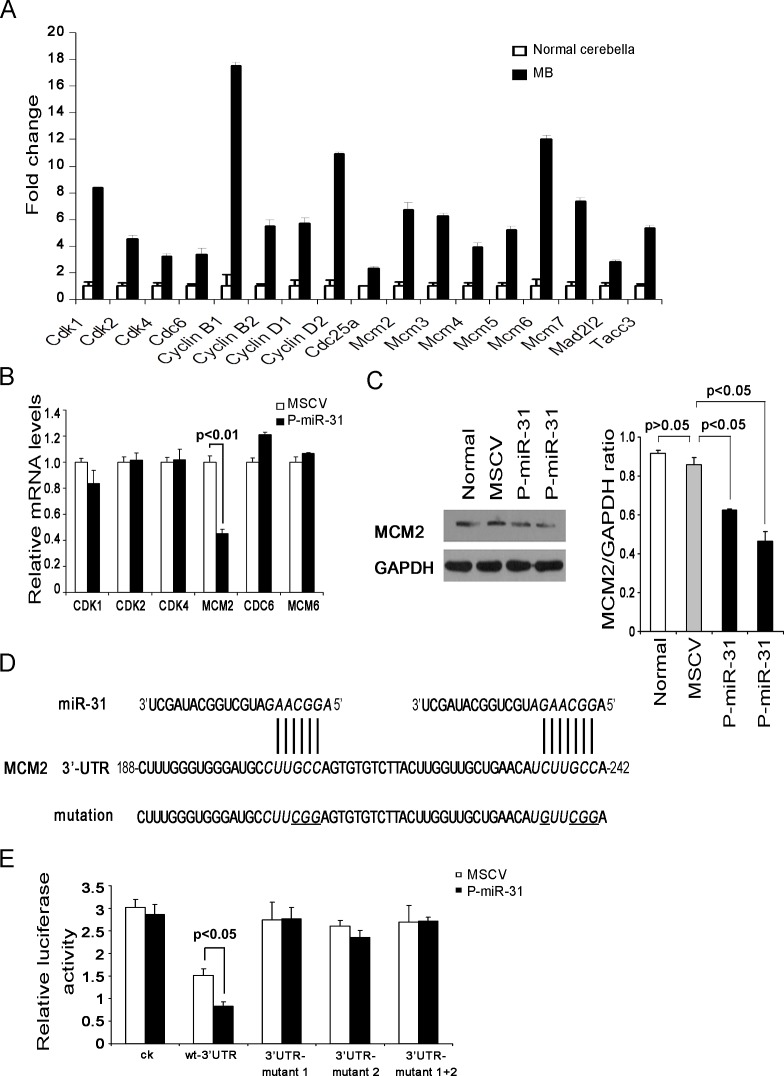
MCM2 is a direct target gene of miR-31 (A) A selected group of cell-cycle associated genes differentially expressed in normal cerebellar and medulloblastoma tissues as determined by microarray analysis. (B) Quantitative RT-PCR analysis of putative miR-31 targets, CDK1, CDK2, CDK4, MCM2, CDC6 and MCM6, in vector control and miR-31 expressing DAOY cells. Data were analyzed according to the comparative *Ct* method, with *GAPDH* as a reference. The expression level in vector control cells was set to 1. (C) Immunoblot analysis of MCM2 protein levels in vector control and two independent pools of miR-31 expressing DAOY cells. GAPDH served as an internal control. The bar graph represents the relative MCM2 band intensity (on right panel). It was calculated as a ratio of MCM2 and GAPDH. (D) Predicted miR-31 target recognition sites in the 3'-UTR of human MCM2. The wild type luciferase reporter was generated with a DNA fragment covering two putative miR-31 binding sites. Mutations in the two miR-31 target sites are underlined. (E) Luciferase assays for the effect of re-expressing miR-31 response on the MCM2 3'-UTR reporters and its mutant variants in DAOY cells. The relative luciferase activity, defined as the ratio of the activity of MCM2 3'-UTR reporter (firefly) to that of the internal control (Renilla), was determined 48 h after transfection, and data representing the average of three independent experiments were shown.

MicroRNAs regulate gene expression by translational suppression or induction of mRNA degradation at target sequences often located in the 3'-UTR. For this reason, global gene expression profiling coupled with bioinformatics analysis of microRNA recognition sequences have been proven effective in identifying target genes. We compared gene expression profiles of *Ptch*^+/-^ mouse medulloblastomas with those of normal cerebella, and found that many cell cycle genes were up-regulated in medulloblastomas based on Gene ontogenic classification (Figure 3A and Supplementary Table 2). Six of this group contain miR-31 recognition sequences in their 3'-UTR, but RT-PCR experiments indicated that only MCM2 was down-regulated in the P-miR-31 DAOY cells (Figure 3B and Table 1). Western analysis also showed marked reduction of MCM2 protein level in the two independent pools of miR-31 expressing DAOY cells (Figure 3C). The 3'-UTR of MCM2 contains two closely situated putative miR-31 recognition sequences (Figure 3D). When attached to the end of a luciferase reporter, the MCM2 3'-UTR repressed the luciferase activity in the P-miR-31 DAOY cells but not in the control MSCV DAOY cells (Figure 3E), presumably due to microRNA-mediated translational repression or mRNA degradation. However, if either one of these two recognition sequences or both were mutated, the MCM2 3'-UTR was no longer capable of repressing luciferase activity (Figure 3E). Thus, MCM2 is a direct target of miR-31.

**Table 1:** Putative targets of miR-31 predicted by MiRanda, Targetscan and PITA

Gene	Accession Number	Gene Expression Array	Computer Predictions		
		Fold change	MiRanda	Targetscan	PITA
CDK1	NM_033379	8.413	+	+	+
CDK2	NM_001798	4.548	+	-	+
CDK4	NM_000075	3.260	-	-	+
MCM2	NM_004526	6.737	+	+	+
CDC6	NM_001254	3.374	+	+	+
MCM6	NM_005915	12.002	+	-	+

### MiR-31 regulates MCM2 function at the DNA replication origin

**Figure 4:**
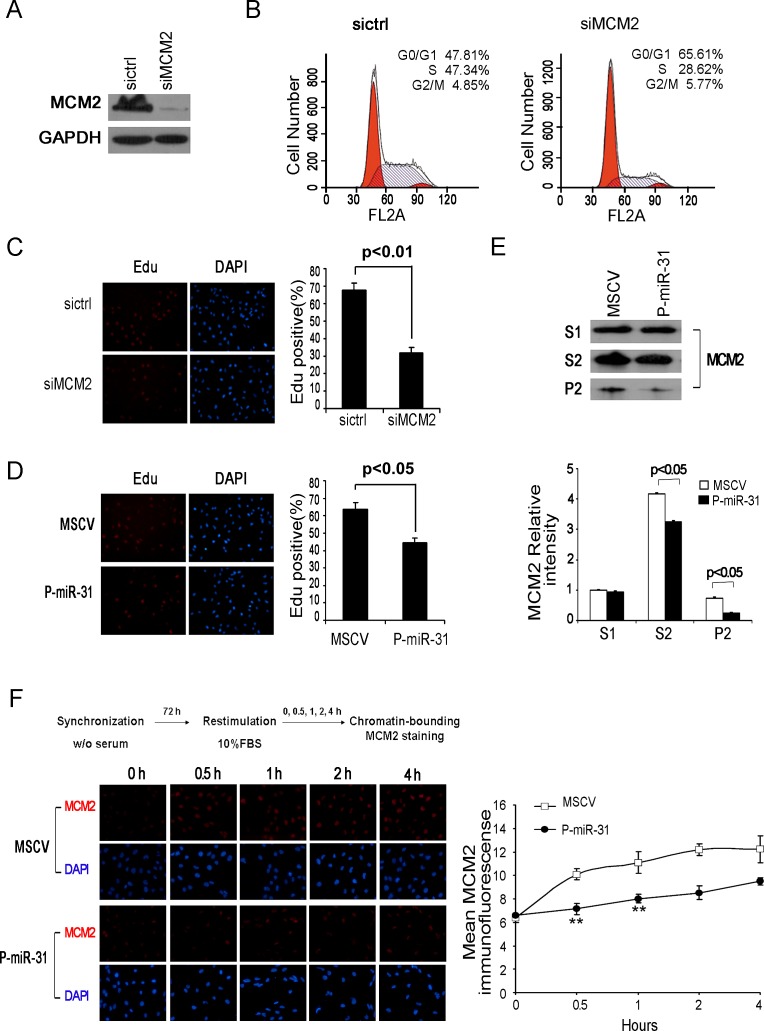
MiR-31 regulates MCM2 function at the DNA replication origin (A) DAOY cells, transfected with MCM2 siRNA for 48 h, were harvested and the whole-cell extracts were immunoblotted with antibody against MCM2. GAPDH was shown as a loading control. (B) Flow cytometric histograms of propidium iodide-stained DAOY cells transfected with control siRNA, MCM2 siRNA for 48 h. The percentages of cells in G1, S and G2 phase of the cell cycle were determined by analysis with the Multicycle computer software. (C) Edu incorporation assays of DAOY cells transiently transfected with control siRNA or siMCM2, and (D) stable miR-31-expressing DAOY cells. The percentage of Edu positive cells was blindly calculated with counting several nonoverlaping fields. Values are means ± SD. (E) Western analysis of chromatin-bound MCM2. DAOY cells stably expressing miR-31 were fractionated into Triton-soluble (S1 fraction) and –insoluble fractions by CSK/0.5% Triton X-100 buffer. The latter fractions were extracted with DNase I and salt. The supernatant (S2 fraction, containing DNase-released chromatin-associated proteins) and pellet (P2, containing insoluble, cytoskeletal, and nuclear matrix proteins) were collected for immunoblotting assay. Bottom panel shows the relative intensity of MCM2 in S1, S2 and P2 fractions. The expression level of Triton-soluble MCM2 in MSCV cells was set to 1. (F) Fluorescence detection of chromatin-bound MDM2 in synchronized DAOY cells. Stabe DAOY cells overexpressing miR-31 were synchronized at the G0/G1 boundary by serum deprivation and thereafter were released into fresh medium containing 10% serum. At the times indicated after the release, cells were extracted with CSK/0.5% Triton X-100 buffer before fixation and then stained with α-MCM2 (red) and DAPI (blue). Right panel shows the average MCM2 immunofluorescence per nucleus of vector control and miR-31 expressing DAOY cell nuclei at the G1/S transition. Data represent averages of three independent experiments in triplicate.

MCMs are licensing factors for eukaryotic DNA replication. MCM2 is one of the six MCMs that form a pre-replication complex involved in both DNA-replication initiation and elongation. Indeed, flow cytometric analysis of the cell cycle profile following knockdown of MCM2 by siRNA indicated that this treatment arrested DAOY cells at the G1/S boundary (Figure 4A and 4B), consistent with the role of MCM2 in DNA replication. To determine if miR-31 regulates MCM2 function, we compared the effects of MCM2 knockdown and miR-31 re-expression on the ability of DAOY cells to incorporate fluorescence dye, EdU, which is a measure of DNA synthesis. The results showed that both treatments reduced the rate of DNA synthesis to comparable levels (Figure 4C and 4D). Thus, restoration of miR-31 expression in DAOY cells has an equivalent impact on DNA synthesis as knockdown of MCM2.

The MCM pre-replication complex has a ring structure and is associated with the chromatin at the forks of DNA replication origin [31, 32]. After initial separation of the nuclear (P1 fraction) and the cytoplasmic fractions (S1 fraction), the chromatin-bound MCM complex (S2 fraction) can be released from the nuclear matrix (P2 fraction) into a soluble form by DNase I treatment and salt extraction. Following this procedure, we found that the amounts of the soluablized chromatin-bound MCM2 in the S2 fraction and the nuclear matrix associated MCM2 in the P2 fraction in P-miR-31 DAOY cells were significantly lower than those in the control MSCV DAOY cells (Figure 4E), indicating that miR-31 re-expression affects the effective concentration of MCM2 in the chromatin.

Because overexpression of miR-31 showed decreased proliferation and G1/S delay, it is probable that the reduced chromatin-bound MCM2 levels are entirely attributable to growth defects. To test this, we assessed the levels of chromatin-bound MCM2 in P-miR-31 DAOY cells during G1/S. DAOY cells were synchronized at G0 by serum deprivation for 3 days, and then released into different stages of the cell cycle by addition of fresh medium. Consistent with previous reports [33], chromatin association of MCM2 increased dramatically during G1/S in both vector control and miR-31 expressing DAOY cells (Figure 4F). In contrast, quantification of the average MCM2 immunofluorescence of the nucleus revealed a significant difference between control and miR-31 expressing cell nuclei at the G1/S transition, with the mean value of miR-31 expressing cells nuclei being much lower than that of vector carrying cells nuclei, especially in the early phases of stimulation (Figure 4F, right panel).

## DISCUSSION

In the present study, we describe that miR-31 is significantly under-expressed in the *Ptch*^+/-^ mouse medulloblastoma model and human medulloblastoma cell lines, suggesting that aberrant miR-31 expression might be a common event in the medulloblastoma tumorigenesis. Our findings are in line with a previously published microRNA study in human medulloblastoma samples [28]. DAOY cells lack a functional TP53 [34], but our data showed that restoring miR-31 expression is sufficient to inhibit their growth. Since up to 40% of medulloblastomas have a dysfunctional TP53 pathway and are resistance to conventional chemotherapy [35], this observation may open a new route for treating medulloblastoma.

The miR-31 gene is located within the first intron of a previously uncharacterized gene, LOC554202, at 9p21.3, adjacent to tumor suppressor p16*^CDKN2A^*. Concurrent deletion of both miR-31 and p16*^CDKN2A^* was observed previously in multiple melanomas and urothelial carcinomas in the bladder [29, 30]. In the present report, we performed genomic PCR detection of regions surrounding the p16*^CDKN2A^* locus and found that miR-31 was deleted along with p16*^CDKN2A^* in DAOY cells. It is possible that the deletion of miR-31 in DAOY cells was consequential to the deletion of p16*^CDKN2A^*, which is a frequent event associated with immortalization. However, several recent reports showed that the miR-31 promoter was epigenetically inactivated through DNA hypermethylation in CpG islands in primary breast and prostate cancer tissues, independent of the status of the p16*^CDKN2A^* locus[36, 37]. These new data suggest that miR-31 may possess tumor suppressor functions of its own. Indeed, our results indicated that restoring miR-31 expression strongly suppresses the ability of DAOY cell to grow, form anchorage dependent colonies in vitro, as well as xenograft tumors in nude mice. Since DAOY cells are TP53 negative, the tumor suppressor function of miR-31 is likely mediated by mechanisms other than apoptosis.

We compared expression profiles between medulloblastoma and normal cerebellar tissues by microarray analysis, and found that many cell cycle-associated genes were ostensibly up-regulated in medulloblastomas. Through bioinformatics and expression analysis, we identified MCM2 as a putative target of miR-31. In our study, a significant down-regulation of MCM2 transcript and protein was observed after miR-31 overexpression in DAOY cells. Luciferase reporter assay further confirmed that miR-31 specifically acts on MCM2 3'-UTR, via the miR-31 binding site, of MCM2. MCM2 is one of the six highly conserved proteins that form a double hexameric MCM complex at the DNA replication origins in eukaryotes. The MCM complexes are key components of the pre-replication apparatus and may be involved in the formation of replication forks for recruiting other DNA replication related proteins [31, 32]. We also investigated whether regulation of MCM2 expression by miR-31 influences the expression of other members of the MCM complex. Our data showed that expression of MCM6, the only MCM complex subunit that possesses miR-31 recognition sites in its 3'-UTR, was not changed after miR-31 overexpression. Moreover, there was no difference in expression levels of other MCMs between miR-31-overexpressing cells and control cells (data not shown).

As described earlier, MCM2-7 complex is associated with chromatin and involved in DNA replication [31, 32]. The complex is assembled in the cytoplasm, and imported into the nucleus during the G1 phase [38]. The import process is dependent upon synergistic nuclear localization signals present on MCM2 and MCM3 [39]. During the S, G2 and M phases, MCM complexes are excluded from the nucleus to avoid over-firing at the DNA replication origin, which could cause a catastrophe to the genomic integrity [38]. The export of MCM complexes is dependent upon MCM3 that has a nuclear export signal [39]. We examined the levels of chromatin-bound MCM2 in miR-31 expressing DAOY cells using immunoblotting analysis and immunofluorescence staining, and found that restoring miR-31 expression caused a marked decrease in the levels of chromatin-bound and nuclear matrix-bound MCM2, but those of the cytosolic fractions were not affected at all. Consistent with previous reports [33], the level of chromatin-bound MCM2 gradually increases after release from serum-starvation block, reflecting the resumed DNA replication activities in the G1 phase. During the process, however, the rate of MCM accumulation in miR-31 expressing DAOY cells was markedly retarded compared to that of the vector control cells. Intriguingly, we found that, like that of MCM2, the level of chromatin-bound and nuclear matrix-bound MCM7 was also reduced in miR-31 expressing DAOY cells compared to the vector control cells (data not shown). In the observation of parallel MCMs decrease, the general assumption is that there is hexamer destabilization or impaired MCM chromatin loading followed by degradation of monomers. Although others also reported that knockdown of MCM2, MCM3, MCM5 in human cells decreased the amount of other chromatin-bound MCMs [40, 41], the mechanisms for coordinated regulation and its triggers remain unknown.

**Figure 5:**
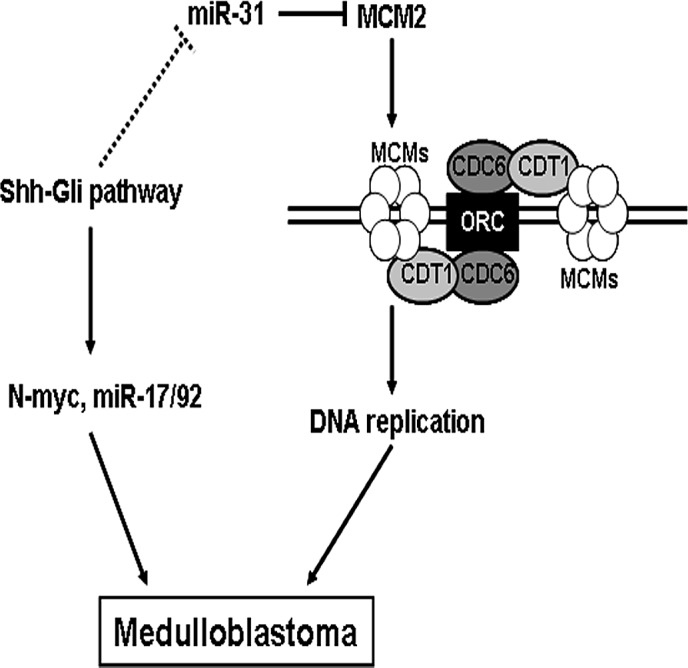
A model for the role of miR-31 in suppressing medulloblastoma tumorigenesisi Lines with arrowheads denote stimulatory pathways, whereas lines with flat end denote inhibitory pathways. The dotted line refers to an undefined pathway.

On the basis of our results, we propose a model for the role of miR-31 in medulloblastoma (Figure 5). During the medulloblastoma tumorigenesis process, genetic or epigenetic inactivating mechanisms cause a shutdown of miR-31 expression, which leads to an enhanced expression of its downstream target gene MCM2. Elevated chromatin-bound MCM2 accelerates DNA replication and facilitates malignant transformation. Activation of Shh pathway, such as heterozygous deletion of Ptch, could cause medulloblastoma [42]. Gli transcription factors are critical effectors of the Shh signaling pathway and regulate expression of multiple targets, including N-myc and miR-17/92 [19, 43]. Toward that end, we also identified Gli consensus DNA-binding sequences in the promoter region of miR-31 gene, suggesting a potent connection between miR-31 and Shh pathway in medulloblastoma.

## MATERIALS AND METHODS

### Cell culture, transfection and synchronization

The human medulloblastoma cells (DAOY and D283), non-small cell lung carcinoma cells (A549 and NCI-H322M), breast cancer cells (MCF-7 and MDA-MB-231), rhabdomyosarcoma cells (RD and RH30), prostate cancer cells (PC3 and DU145), hepatocellular carcinoma cells (HEPG2 and HEP3B), and human embryonic kidney cells (HEK 293) were purchased from ATCC, and cultured in DMEM medium (GIBCO) supplemented with 10% FBS according to the supplier's recommendations. Specific siRNA against human MCM2 and negative control siRNA were purchased from Ribobio. In each case, 100 nM oligonucleotides were used in transient transfection with Oligofectamine (Invitrogen) as a delivery agent. After 24 h, cell cycle and EdU incorporation assay were performed. For synchronization, DAOY cells were synchronized at the G0 phase by serum starvation for 72 h, released into cell cycle by addition of fresh medium with 10% serum, and then harvested at the indicated time points after release.

### Stable cell lines

A 151 bp human genomic DNA fragment containing precursor miR-31 was isolated from HEK293 cells by PCR amplification using PrimerStar DNA Polymerase (Takara) and subcloned into the BamHI and XhoI sites of pRetro-MSCV-H1G vector. All primers used are listed in the Supplementary Table 3. The plasmid was verified by DNA sequencing. After 2 weeks of selection with puromycin, clonal DAOY cells were picked from isolated foci using cloning rings and expanded into separate lines.

### Cell line authentication

STR DNA profiling analysis was used for Cell line authentication. NJYK-003 was found with 100% matched to the Daoy cell line in the ATCC and DSMZ databases; 100% matched cell line named D283 Med, NCI-H322M, HepG2, MCF-7 was found in the ATCC,DSMZ or JCRB data bank and no cross-contamination of other human cells was found in this test.

### Animals

The *Ptch*^+/−^mice were purchased from the Jackson Laboratory. Cohorts of mice were observed for tumor formation for 6-8 months after birth, and sacrificed when they showed signs of increased intracranial pressure (ataxia, decreased movement, paresis of hind limbs, enlarged occipital prominence, hunched back, and/or poor grooming). Tumor tissues were carefully separated under a dissecting microscope. Fresh tissues were snap-frozen and stored at −80 °C for later extraction of RNA. For histochemical analysis, animals were anesthetized and perfused transcardially with 4% paraformaldehyde. Paraffin-embedded (5 μm), 4% paraformaldehyde-fixed tissue sections were stained with H&E to evaluate the morphology. BALB/C female athymic mice (4-5 weeks old) were purchased from Model Animal Research Center of Nanjing University. All mice were maintained and bred under specific pathogen-free conditions at Nanjing Medical University. All experiments were undertaken with the approval of Nanjing Medical University Animal Ethics Committee.

### NanoString nCounter assay and data analysis

Expression profiling of miRNA was conducted on 3 medulloblastoma and 3 normal cerebellar tissue samples from Ptch+/- mice using the NanoString nCounter assay (NanoString Technologies, Seattle, USA). The mouse miRNA expression array contains 578 miRNA gene probes using four house-keeping genes(Actb, B2m, Gapdh and Rpl) as the internal controls. The total RNAs from the above samples were hybridized with the capture and reporter probes and incubated overnight at 65°C according to the manufacturer's recommended protocol. The target and probe complexes were washed and immobilized in the cartridge, and the amount of mRNA was quantified in the nCounter Digital Analyzer. The data were normalized to spike-in controls and to the geometrical means of the four house-keeping genes in each hybridization reaction. Data from Nanostring nCounter assays were analyzed using Agilent GeneSpringGX (v.12.0) software. The differences between the means of experimental groups of normalized data were analyzed by an unpaired student *t* test. The miRNAs with statistically significant *p* values (p<0.01) and fold changes (≥3-fold) were selected for further analysis. The signal intensity values and fold changes presented in figures and online supplementary tables are from data normalized to the control gene probes. Microarray Total RNAs of mouse normal cerebellar tissues and medulloblastoma were extracted using TRIzol reagent as recommended by the manufacturer (Invitrogen). Subsequent steps for the hybridization to Agilent mouse whole genome 4 × 44K array were done according to standard Agilent protocols. The array data were analyzed with Agilent feature extraction and GeneSpring GXv7.3.1 software (Agilent Technologies). A gene is considered to be differentially expressed when the change in gene expression between normal cerebellar and MB tissues was greater than 2-fold in the same direction in both replicate experiments.

### Semi-quantitative RT-PCR and quantitative RT-PCR

The reverse transcriptions were carried out using the PrimeScript RT reagent kit (Takara). To detect the expression of mature miR-31 in different cancer cells, the RT product was amplified in conventional PCR using a miR-31-specific forward primer and the universal reverse primer. The products of looped RT-PCR were analyzed in 2% agarose. Samples were normalized to *U6*. Real time PCR was carried out in a SYBR Premix Ex Taq (Takara) in an ABI Prism 7500 Sequence Detection System (Applied Biosystems). The reactions were incubated in a 96-well plate at 95 °C for 5 min, followed by 40 amplification cycles of 95 °C for 15 s and 60 °C for 1 min. Data were analyzed according to the comparative *Ct* method, with *U6* or *GAPDH* as a reference. All PCR reactions were performed in triplicate. All primers used are listed in Supplementary Table 4.

### Genomic PCR

Genomic DNA was extracted from 293 and DAOY cells, and genomic PCR was performed on the p16*^CDKN2A^*-flanking areas. All primers used are listed in Supplementary Table 5. The PCR products were analyzed in 1% agarose.

### Cell viability and colony formation

Cells were plated in 96-well plates at a density of 3 × 10^3^ cells per well. Cell viability was assessed in six consecutive days. MTT solution (5 mg/ml) was added to each well, and the cells were maintained for 4 h at 37 °C. 100 μl dimethyl sulfoxide (DMSO) was then added and the absorbance at 490 nm was measured. For the colony formation assay, 500 cells were plated in a p60 plate and allowed to grow until visible colonies appeared. Colonies were stained with Giemsa and counted.

### Flow cytometric analysis of DNA content

After various treatments as indicated, DAOY cells were harvested by trypsinization, washed twice with PBS, and then fixed in 70% ethanol overnight. Cells were treated with 100 U/ml RNase for 15 min at 37 °C, resuspended and hypotonically lysed in 1 ml of propidium iodide (PI) before subjecting to FACS analysis.

### Edu incorporation assay

After various treatments as indicated, DAOY cells were seeded on coverslips and cultured overnight. On the next day, cells were incubated for 6 h with 10 μM Edu, and fixed in 3.7% formaldehyde followed by Edu detection as described by the manufacturer (Invitrogen).

### Immunoblotting

30 μg proteins were separated by 12% SDS-polyacrylamide gels. Anti-rabbit MCM2 antibody (Epitomics) and anti-GAPDH (Kangchen) were used as the primary antibodies.

### Luciferase reporter plasmids construct

To prepare reporter plasmid for MCM2, a partial 3'-UTR fragment of MCM2 (520 bp), containing two putative miR-31 binding site corresponding to +2873 to +3393 of human mRNA sequence (NM_004526.3), was amplified with primers containing XbaI linker using genomic DNA. PCR product was then cloned into the unique XbaI site downstream of luciferase gene in the pGl3-control vector (Promega), and the resultant plasmid was termed pGl3-MCM2-wt-3'UTR. The mutated plasmids were generated using the QuickChange XL Site-Directed Mutagenesis Kit (Stratagene). All primers used are listed in Supplementary Table 3. DNA sequencing was performed to verify plasmid sequences.

### Luciferase Assays

DAOY cells carrying constitutive miR-31 expressing or control vectors were co-transfected in 24-well plate with 500 ng pGl3-control vector (pGl3-ck) or pGl3-MCM2-wt-3'UTR or mutant reporter plasmids, and 20 ng of pRL-TK plasmid (Renilla) using Lipofectamine 2000. Luciferase activity was measured by Dual-luciferase Reporter Assay System (Promega) at 48 h post-transfection. Transfection efficiency was corrected by normalizing 3'-UTR reporter activity (firefly) to that of Renilla. Each experiment was performed in triplicate and repeated three times.

### *In vivo* xenograft study

Twenty mice were injected subcutaneously with either 3 × 10^6^ control vector (ten animals) or miR-31 expressing DAOY cells (ten animals). The palpable tumor diameters were measured once a week. Tumor volumes were calculated as ab^2^/2 (where a is length and b is cross-sectional diameter).

### Chromatin binding assay

The method of He *et al.* [25] was used, with modifications described by Kannouche *et al.* [26]. Briefly, DAOY cells were lysed for 20 min on ice in cold CSK I buffer (10 mM Pipes, pH 6.8, 100 mM NaCl, 1 mM EDTA, 300 mM sucrose, 1 mM MgCl_2_, 1 mM DTT) supplemented with 0.5% Triton X-100, protease inhibitors (Roche) and 1 mM PMSF. After low-speed centrifugation (500 g, 3 min at 4 °C), the supernatants (S1 fraction), which contains Triton-soluble proteins, were further analyzed. The pellets, which contain chromatin-bound, nuclear matrix-bound, and insoluble proteins, were resuspended in CSK II buffer (10 mM Pipes, pH 6.8, 50 mM NaCl, 300 mM sucrose, 6 mM MgCl_2_, 1 mM DTT), and treated with DNase I for 30 min followed by extraction with 250 mM NH_2_SO_4_ for 10 min at 25 °C. The sample treated with DNase I and salt was then centrifuged at 1200 g for 6 min at 4 °C and the supernatant (S2 fraction, containing DNase I-released chromatin-associated proteins) and pellet (P2, containing insoluble, cytoskeletal and nuclear matrix proteins) were collected. P2 was also resuspended in RIPA buffer. All fractions were analyzed by immunoblotting. For immunofluorescence analysis, synchronized DAOY cells were harvested at the indicated time points and extracted with CSK buffer containing 0.5% Triton X-100 to remove cytosolic and nucleosic proteins but retain the chromatin-bound proteins. Then cells were fixed with 4% paraformaldehyde and stained with monoclony MCM2 antibody with the regular immunofluorescence protocol. To verify DNA content, cells were costained with DAPI.

### Statistic Analysis

All of the data are presented as the means ± S.D.(n=3). We performed statistical analysis by *t* test, and *p*<0.05 was considered statistically significant. *p>0.05,**p<0.05,***p<0.01.

## SUPPLEMENTARY MATERIALS AND TABLES






