# Supplementary material for: MicroRNA-31 suppresses medulloblastoma cell growth by inhibiting DNA replication through minichromosome maintenance 2
Source: Oncotarget. 2014 May 30;5(13):4821–33. doi: 10.18632/oncotarget.2043 (PMC4148102; doi:10.18632/oncotarget.2043)
Supplement: Supplementary file 3 [file oncotarget-05-4821-s003.pdf]

MicroRNA-31 suppresses medulloblastoma cell growth by inhibiting DNA replication through minichromosome maintenance 2

Supplementary Material

Supplementary Table 3 Primer sequences used for vector construct.

Primer Name	Sequence(5'-3')
pre-miR-31-F(BamHI)	GAAGGATCCTAGTATTCTCCTGTAACTTGGAAC
pre-miR-31-R(XhoI)	ATACTCGAGGGACTGGCATGCAGGTGGCCATGG
pGl3-MCM2-3'UTR-F	GCTCTAGAGGGTACTAGGGTCAGGGCTTAT
pGl3-MCM2-3'UTR-R	GCTCTAGAACCAAACCGAATGCACAAAT
pGl3-MCM2-3'UTR-Mutant 1-F	GGGTGGGATGCCTTCGGAGTGTGTCTTACTTGG
pGl3-MCM2-3'UTR-Mutant 1-R	CCAAGTAAGACACACTCCGAAGGCATCCCACCC
pGl3-MCM2-3'UTR-Mutant 2-F	GGTTGCTGAACATGTTCGGACCTCCGAGTGCTTTG
pGl3-MCM2-3'UTR-Mutant 2-R	CAAAGCACTCGGAGGTCCGAACATGTTCAGCAACC

Supplementary Table 4 Primer sequences used for RT-PCR and quantification RT-PCR.

Primer Name	Sequence(5'-3')
miRNA reverse primer	GTGCAGGGTCCGAGGT
mmu-miR-31 stem-loop primer	GTCGTATCCAGTGCAGGGTCCGAGGTATTCGCACTGG ATACGACAGCTAT
hsa-miR-31 stem-loop primer	GTCGTATCCAGTGCAGGGTCCGAGGTATTCGCACTGG ATACGACCAGCTA
hsa/mmu-miR-31-F	AGCCGAGGCAAGATGCTGGCA
hsa/mmu-U6-F	CTCGCTTCGGCAGCACATATAC
hsa/mmu-U6-R	GAACGCTTCACGAATTTGCGTG
hsa-CDK1-F	GGGTAGACACAAAACTACAGGTC
hsa-CDK1-R	TCCTGCATAAGCACATCCTGA
hsa-CDK2-F	CCCAGATGAGGTGGTGTGG
hsa-CDK2-R	CCGCTTGTTAGGGTCGTAGTG

hsa-CDK4-F	CTGGTGTTTGAGCATGTAGACC
hsa-CDK4-R	AAACTGGCGCATCAGATCCTT
hsa-MCM2-F	CCGTGACCTTCCACCATTTGA
hsa-MCM2-R	GGTAGTCCCTTTCCATGCCAT
hsa-CDC6-F	GCTGTTGAACTTCCCACCTT
hsa-CDC6-R	TTGCGAACATCTCCTGAAAC
hsa-MCM6-F	CTGCTGTGATGAGGTCCAAC
hsa-MCM6-R	GCACCCTCATCTACCTCCAT
hsa-GAPDH-F	ATCATCCCTGCCTCTACTGG
hsa-GAPDH-R	GTCAGGTCCACCACTGACAC

Supplementary Table 5 Primer sequences used for mapping deletions in 9p21.3.

**Primer Name**	**Sequence(5'-3')**
p16^{CDKN2A}-F	GAATCCCGTAGCTTCCCTAC
p16^{CDKN2A} -R	CGGGTCCCGATTTAGAAGG
9p minus 1Mbps-F	CCTGCTGTGCACTAGATCTC
9p minus 1Mbps-R	AGGGCTCCCACTGATTCTG
9p plus 1Mbps-F	GTTATGCTGCATTCCAGATGG
9p plus 1Mbps-R	GTAATGCTTCCAGGTCTATGC
CDK1-F	GTTTCCATATGTTATATCAACAG
CDK1-R	GAGCCTTTTTAGATGGCTGCTAA
